# Surgical salvage intervention for a migrated metal stent during endoscopic ultrasound-guided hepaticogastrostomy

**DOI:** 10.1055/a-2208-5293

**Published:** 2023-12-11

**Authors:** Rei Ryozawa, Hiroyuki Kojima, Kenjiro Yamamoto, Yuichi Nagakawa, Takasyoshi Tsuchiya, Shuntaro Mukai, Takao Itoi

**Affiliations:** 1 13112 Department of Gastroenterology and Hepatology, Tokyo Medical University, Tokyo, Japan; 2 13112 Department of Gastroenterology and Hepatology, Tokyo Medical University, Tokyo, Japan; 3 13112 Department of Gastrointestinal and Pediatric Surgery, Tokyo Medical University, Tokyo, Japan

Proximal metal stent migration during or after endoscopic ultrasound-guided hepaticogastrostomy (EUS-HGS is a serious adverse event, causing severe biliary peritonitis 1 . Although it is sometimes possible to endoscopically remove a migrated stent into the abdominal cavity, most cases require open salvage surgery 2 3 4 5 . Herein, we report a case of successful minimally invasive laparoscopic repair of a migrated metal stent guided by a guidewire left at the gastric puncture site.

A 16-year-old girl who underwent extrahepatic duct excision and hepaticojejunostomy for congenital bile duct dilatation 15 years ago presented with cholangitis 5 years ago. An abdominal computed tomography (CT) scan revealed dilatation and infection of the cystic bile duct in left lobe of the liver ( Fig. 1 ), and her symptoms improved with antibiotic treatment. With increased frequency of recurrent epigastric and back pain, she was referred for endoscopic treatment of recurrent cholangitis in our hospital. Balloon endoscopy-assisted endoscopic retrograde cholangiopancreatography (ERCP) showed no stenosis at the hepaticojejunostomy, but the tortuous and narrow intrahepatic bile duct communicated with a cystically dilated bile duct. Since a guidewire could not pass the narrow part of bile duct ( Fig. 2 ), EUS-HGS was conducted.

**Fig. 1:**
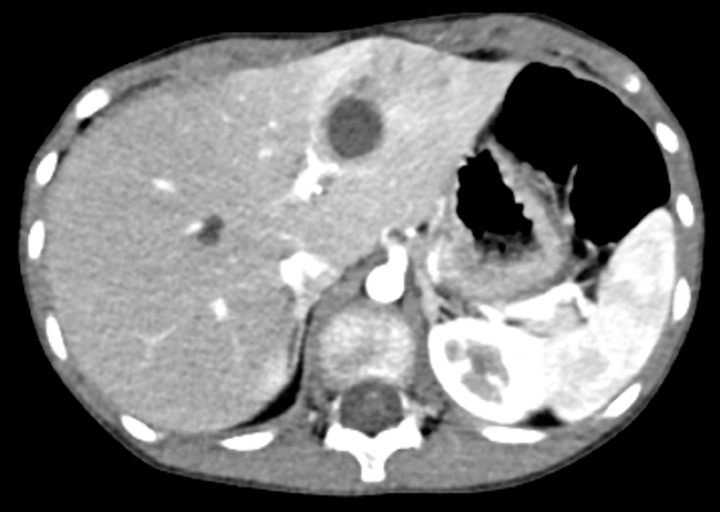
The abdominal contrast-enhanced computed tomography revealed the cystically dilated bile duct in the left lobe of the liver.

**Fig. 2:**
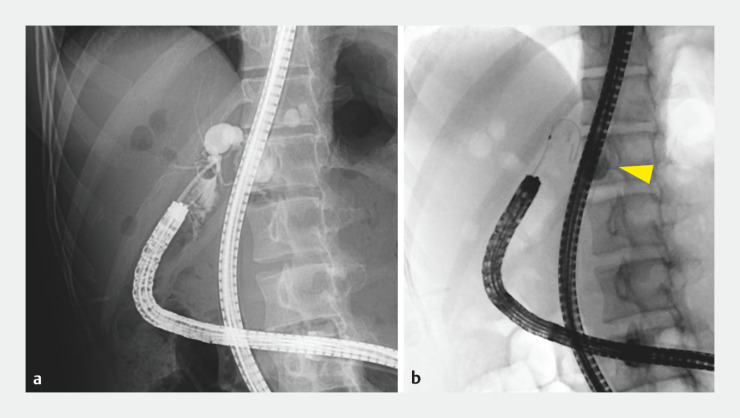
**a** Balloon enteroscopy-assisted endoscopic retrograde cholangiopancreatography showed the tortuous and narrow intrahepatic bile duct communicated with the cystically dilated bile duct. **b** A guidewire could not pass the narrow part of the bile duct, and the cholangiogram showed a cystically dilated intrahepatic bile duct (yellow arrowhead).

After tract dilation, we tried to place a 6-mm fully covered self-expandable metal stent (SEMS). However, the edge of SEMS on the gastric side migrated into the abdominal cavity. After unsuccessful attempts to retrieve the stent endoscopically via the puncture site, surgical intervention was deemed necessary. We placed two guidewires orally from the gastric fistula into the abdominal cavity so that surgeons could find the gastric fistula ( Fig. 3 ) and return the SEMS to the stomach under laparoscopy. Using laparoscopy, the stent was retrieved using forceps and guided back into the stomach ( Fig. 4 , Video 1 ). Using this method, migrated SEMSs that are not endoscopically retrievable can be repaired in a minimally invasive surgical intervention.

**Fig. 3:**
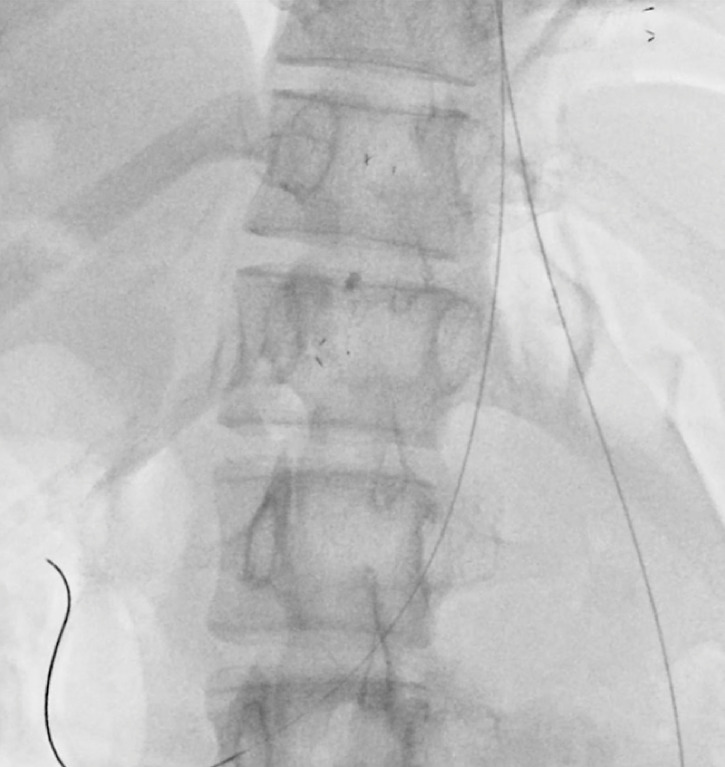
Two guidewires were left in place in the abdominal cavity as a surgical landmark from the gastric fistula.

**Fig. 4:**
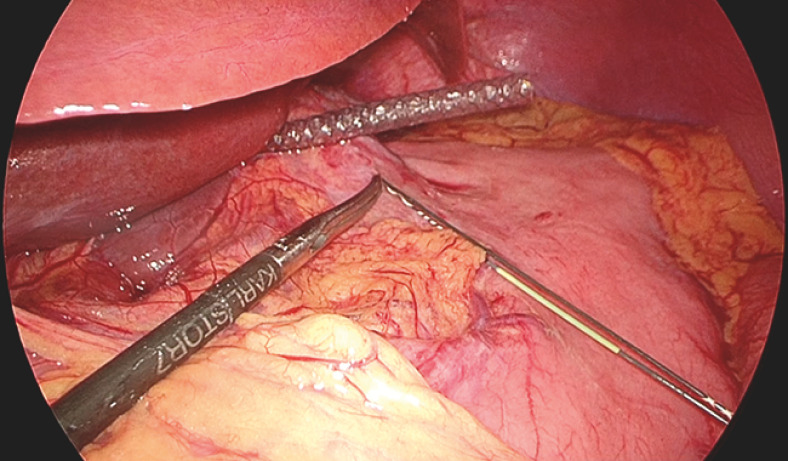
A laparoscopic image showed the gastric fistula owing to the landmark guidewire, and the self-expandable metal stent could be repositioned into its intended location.

Surgical salvage intervention for a migrated metal stent during endoscopic ultrasound-guided hepaticogastrostomy.Video 1

Endoscopy_UCTN_Code_CPL_1AL_2AD

## Notes

Endoscopy E-Videos https://eref.thieme.de/e-videos
*E-Videos* is an open access online section of the journal *Endoscopy* , reporting on interesting cases and new techniques in gastroenterological endoscopy. All papers include a high-quality video and are published with a Creative Commons CC-BY license. Endoscopy E-Videos qualify for HINARI discounts and waivers and eligibility is automatically checked during the submission process. We grant 100% waivers to articles whose corresponding authors are based in Group A countries and 50% waivers to those who are based in Group B countries as classified by Research4Life (see: https://www.research4life.org/access/eligibility/ ). This section has its own submission website at https://mc.manuscriptcentral.com/e-videos .
